# Supplementary material for: Ambiental Factors in Parkinson’s Disease Progression: A Systematic Review
Source: Medicina (Kaunas). 2023 Feb 5;59(2):294. doi: 10.3390/medicina59020294 (PMC9962232; doi:10.3390/medicina59020294)
Supplement: Supplementary file 1 [file medicina-59-00294-s001.zip › medicina-2114556-supplementary.pdf]

Supplementary material

Table S1. Search strategy.

**MEDLINE**: ("PM2.5" "PM10" OR "PM(10)" OR "fine particulate matter" OR "fine particles" OR "nitrogen oxides" OR "nitrogen dioxide" OR ''NO'' OR "NO2" OR '' Sulfate'', OR "carbon monoxide" '' CO'', "air pollution" OR "air pollutants'' or "humidity", "Temperature", or ''UV light'' [mesh]) AND (parkinson disease[mesh] OR parkinson*) **4.609 STUDIES**
**CINAHL**: ((PM2.5 OR " fine particulate matter *" OR (MH " fine particles ") OR'NO'' OR '' Sulfate'', OR '' CO'', "air pollution" OR "air pollutants'' OR "humidity", "Temperature", OR "UV light'') AND ((Parkinson disease) OR (MH " parkinson ")) **68 STUDIES**
**PsyclinINFO** : ((PM2.5" "PM10" OR "PM(10)" OR "fine particulate matter" OR "fine particles" OR "nitrogen oxides" OR "nitrogen dioxide" OR ''NO'' OR "NO2" OR '' Sulfate'', OR "carbon monoxide" OR'' CO'', "air pollution" OR "air pollutants'' or "humidity", "Temperature", or ''UV light'' *") AND ((parkinson disease) OR (parkinsonian*) OR (PD) OR (Parkinson progression*) **32 STUDIES**
**Scopus**: TITLE-ABS ("PM2.5" OR "fine particulate matter" OR "fine particles" OR "nitrogen oxides" ''NO'' OR '' Sulfate'', OR ''carbon monoxide'' OR '' CO'', "air pollution" OR "air pollutants'' or "humidity", "Temperature", or "UV light') AND (''parkinson disease'' OR '' parkinson'') **69 STUDIES**
**Web of Science**: (PM2.5) or (fine particulate matter) or (NO) or (Sulfate) or (CO), or (air pollution) or (air pollutants) or (humidity) or (Temperature), or (UV light) AND (Parkinson disease) **3.776 STUDIES**
**EMBASE**: (exp Parkinson disease/ or Parkinson*.mp. or Parkinsonian.mp. or Parkinsonism.mp. or Parkinson/s) and (exp fine particulate matter / or exp PM2.5/ or exp CO/ or exp or exp nitrogen oxides/ or exp nitrogen dioxide/ or exp NO/ or exp NO2 or air pollution*.mp. or pollutant*.mp. or humidity.mp. or Temperature.mp. or UV light.mp.) **4.662 STUDIES**
